# Botswana should consider expansion of free antiretroviral therapy to immigrants

**DOI:** 10.1002/jia2.25328

**Published:** 2019-06-12

**Authors:** Daniel J Escudero, Tafireyi Marukutira, Alethea McCormick, Joseph Makhema, George R Seage

**Affiliations:** ^1^ Department of Epidemiology Harvard T.H. Chan School of Public Health Boston MA USA; ^2^ Department of Public Health Burnet Institute Melbourne Australia; ^3^ School of Public Health and Preventive Medicine Monash University Melbourne Australia; ^4^ Botswana Harvard AIDS Institute Partnership Princess Marina Hospital Gaborone Botswana

**Keywords:** policy, ARV, HIV care continuum, key and vulnerable populations, linkage to care, public health

Botswana has among the highest level of HIV viral suppression globally, yet HIV incidence remains > 1% per year in adults aged 15 to 49 1, 2. Although causes of this continued elevated incidence have been postulated, a firm understanding remains elusive, especially in the presence of a highly successful HIV treatment programme in Botswana 1. Although Botswana provides free antiretroviral therapy (ART) for all citizens living with HIV through its national HIV programme, the first free national ART programme in sub‐Saharan Africa, non‐citizen immigrants (documented/undocumented) are currently ineligible for treatment within the national programme. Documented refugees living with HIV in camps do have free access to ART as long as they remain within the confines of the camp. Private HIV treatment is available, but remains prohibitively expensive for many non‐citizens. In addition to gaps in treatment coverage among men and young people 3, the lack of free treatment for non‐citizens may contribute to elevated HIV incidence in Botswana, as suggested by research in other settings 4.

There is precedent for providing government‐sponsored HIV treatment to non‐citizens in Botswana. In 2014, a court ruling found that denying non‐citizens in prison access to ART violated their right to receive basic health services, as guaranteed by the Botswana Constitution 5. This ruling, however, has not affected the eligibility of non‐citizens outside of prison. Despite generally accepted international obligations under human rights law to provide access to ART to non‐citizens as a means to ensure health services without discrimination 6, Botswana has yet to expand eligibility for those who lack citizenship. Becoming a Botswana citizen is not easy for immigrants, it takes a waiting period of up to 10 years to naturalize, a process which has no guarantees.

Only citizens are accounted for in national estimates of people living with HIV, HIV incidence and treatment outcomes, leaving the impact of the HIV epidemic among immigrants largely hidden. Understanding the HIV epidemic in the immigrant population may inform considerations in expanding treatment coverage, and may ultimately help Botswana achieve greater viral suppression, and reduce HIV incidence throughout the population.

As Botswana considers joining regional neighbours such as South Africa and Lesotho in developing national HIV treatment policies that include non‐citizens 7, it should consider the available empirical data regarding HIV care among non‐citizens, and identify critical gaps in knowledge. Along with learning from recent policies in countries supporting HIV care to Venezuelan migrants (Brazil, Colombia) 8, we believe there are four essential questions that must be addressed to understand the impact an ART coverage expansion may have on a nation‐wide scale:

1. How many non‐citizen immigrants reside in Botswana?
2. What is the HIV prevalence in this population, and what proportion of non‐citizens living with HIV are on ART?
3. What are the barriers to HIV testing, linkage‐to‐care or retention, and viral suppression among non‐citizen, and how do these compare to citizens?

To encourage thought on the subject, we performed a preliminary review of published literature addressing each of these essential questions. Broadly, we found the published literature on these questions far too limited to properly examine an important change in national HIV treatment policy. Here, we summarize the results of the four questions posed, and identify the largest gaps within each question. Although some data sources distinguish between the documentation status of non‐citizens, this is non‐universal so unless otherwise stated, we refer here to all non‐citizens.

## 1 Non‐citizen immigrant population size

Two published reports were available to estimate the number of non‐citizens in Botswana. The United Nations estimated that 166 thousand non‐citizens resided in Botswana in 2017, about half of whom were from South Africa, Zimbabwe and Zambia, countries with high HIV prevalence 9. Data from the 2011 National Population and Housing Census, the most recent available, suggest that 112 thousand residents (5.5% of the population) reported foreign citizenship 10. Most of the 17,000 who had taken up residence in 2010 reported Zimbabwean nationality. Importantly, many foreign nationals may have been excluded from the Census’ data collection, or failed to report citizenship status indicating potential underestimation of the immigrant population.

## 2 HIV prevalence and ART among those living with HIV

Data from the Botswana Combination Prevention Project (BCPP) suggested that within 30 rural and peri‐urban communities throughout Botswana, the prevalence of HIV among citizens and non‐citizens was approximately equal (20% vs. 22% respectively) 11. However, non‐citizens were significantly less likely to be aware of their positive HIV status (37% vs. 84%, *p *<* *0.001) 11. The BCPP did not include urban populations, and data for non‐citizen HIV prevalence for the largest cities (e.g. Gaborone, Francistown) were not found.

The BCPP also found that only 29% of non‐citizens with known HIV‐positive status were enrolled on ART, compared to 71% of citizens (*p *<* *0.001). These results, however, are still limited by their lack of data from urban settings. The only published data available highlighting ART access and treatment outcomes in an urban setting are from the Maipelo Trust clinic in Gaborone, which reported slower ART initiation, fewer viral load tests, reduced viral suppression and higher mortality among non‐citizens compared with citizens 12.

## 3 Barriers to treatment and retention

Migrant populations throughout the world often experience stigma while accessing health services, with Botswana serving as no exception. Immigrants fleeing economic collapse in Zimbabwe bear much of this burden, as they are frequently the target of xenophobia and stigma from Batswana 13. The macroscopic impact of this stigma has not been demonstrated at a national level, but studies have provided reports of limited access to health care services generally, and for HIV treatment. A 2010 study among 137 non‐citizens found that 53% were afraid of referral to police or immigration authorities if they were to seek health services 14. A 2003 study identified travel/migration as a significant barrier to maintaining ART adherence among patients living with HIV at three private clinics in Gaborone and Francistown 15. Undocumented non‐citizens may face even greater challenges in accessing care: undocumented individuals are often forced to accept employment far below their qualifications (decreasing income or agency) 16, and may avoid hospitals or health centers where police are likely to patrol, increasing risk of deportation 17.

Substantial research is needed to inform potential expansions in non‐citizen testing and treatment coverage. Data may be needed prior to significant policy changes since Botswana already self‐funds at least two‐thirds of its HIV response, and further strain on the country's programme capacity may be detrimental without increased donor input 18. This research should be nationally‐representative and address the extent of disease burden in the migrant population, and the population‐level benefits of viral suppression in vulnerable migrants. Policy decisions should also consider how to ensure undocumented non‐citizens may share in the benefit of treatment expansion. Preliminary review of these four important questions confirms that the HIV epidemic in this vulnerable population remains largely hidden, and its impact on the overall HIV epidemic in Botswana cannot be known without further study. Furthermore, the impact that expanded coverage may have on overall HIV incidence will require even further investigation into long‐term HIV treatment outcomes and antiretroviral resistance among immigrants, as well as patterns of sexual mixing between migrant and citizen communities.

## Competing interests

The authors declare they have no conflicts of interest.

## Authors’ contributions

DJE and TM performed the literature review, drafted the original manuscript and revised the manuscript based on peer review. DJE, TM, AM, JM and GRS evaluated the results of the literature review and provided critical edits to the manuscript. All authors approved the final manuscript.
